# Intimate partner violence and pregnancy spacing: results from a meta-analysis of individual participant time-to-event data from 29 low-and-middle-income countries

**DOI:** 10.1136/bmjgh-2017-000304

**Published:** 2018-01-13

**Authors:** Lauren Maxwell, Arijit Nandi, Andrea Benedetti, Karen Devries, Jennifer Wagman, Claudia García-Moreno

**Affiliations:** 1 Epidemiology, Biostatistics, and Occupational Health, McGill University, Montréal, Quebec, Canada; 2 Institute for Health and Social Policy, McGill University, Montréal, Quebec, Canada; 3 Department of Global Health and Development, Social and Mathematical Epidemiology Group and Gender Violence and Health Centre, London School of Hygiene & Tropical Medicine, London, UK; 4 Division of Global Public Health, Department of Medicine Central Research Services Facility (CRSF), University of California, San Diego, California, USA; 5 Department of Reproductive Health and Research, World Health Organization, Geneva, Switzerland

**Keywords:** Intimate partner violence, maternal and child health, low-and-middle-income countries, interpregnancy intervals, unintended pregnancy, birth spacing, survival analysis, Cox proportional hazards models, meta-analysis

## Abstract

**Introduction:**

Inadequately spaced pregnancies, defined as pregnancies fewer than 18 months apart, are linked to maternal, infant, and child morbidity and mortality, and adverse social, educational and economic outcomes in later life for women and children. Quantifying the relation between intimate partner violence (IPV) and women’s ability to space and time their pregnancies is an important part of understanding the burden of disease related to IPV.

**Methods:**

We applied Cox proportional hazards models to monthly data from the Demographic and Health Surveys’ Reproductive Health Calendar to compare interpregnancy intervals for women who experienced physical, sexual and/or emotional IPV in 29 countries. We conducted a one-stage meta-analysis to identify the periods when women who experienced IPV were at the highest risk of unintended and incident pregnancy, and a two-stage meta-analysis to explore cross-country variations in the magnitude of the relation between women’s experience of IPV and pregnancy spacing.

**Results:**

For the one-stage analysis, considering 52 959 incident pregnancies from 90 446 women, which represented 232 394 person-years at risk, women’s experience of IPV was associated with a 51% increase in the risk of pregnancy (95% CI 1.38 to 1.66), although this association decreased over time. When limiting our inference to unintended pregnancies that resulted in live births, women’s experience of IPV was associated with a 30% increase in the risk of unintended pregnancy (95% CI 1.25 to 1.34; n=13 541 pregnancies, 92 848 women, 310 319 person-years at risk). In the two-stage meta-analyses, women’s experience of IPV was associated with a 13% increase in the probability of incident pregnancy (95% CI 1.07 to 1.20) and a 28% increase in the likelihood of unintended pregnancy (95% CI 1.19 to 1.38).

**Conclusions:**

Across countries, women’s experience of IPV is associated with a reduction in time between pregnancies and an increase in the risk of unintended pregnancy; the magnitude of this effect varied by country and over time.

**Key questions**

**What is already known about this topic?**

- Research from high-income countries and from cross-sectional data from several low-income and middle-income countries suggests that women who experience intimate partner violence may be less able to space their pregnancies than women who do not.
- This is the first analysis to estimate the relation between women’s experience of intimate partner violence and pregnancy spacing that uses retrospective information on the date of violence initiation rather than a concurrent measure of violence for most of the 29 low-and-middle-income countries included in this analysis.
- In contrast to prior analyses in low-income and middle-income countries that are limited to live births, this analysis includes information on the spacing of all pregnancies, whether or not they resulted in live births, which may provide more accurate estimates given that partner violence is associated with pregnancy termination.

**What are the new findings?**

- Considering population-representative data from 29 countries, women’s experience of intimate partner violence was associated with a 51% increase in the risk of pregnancy (95% CI 1.38 to 1.66) and a 30% increase in the risk of unintended pregnancies that resulted in live birth.

**Recommendations for policy**

- Because of the centrality of pregnancy spacing to maternal and child health, results from this analysis may help inform estimates of the burden of disease related to women’s experience of intimate partner violence.

## Introduction

Women’s ability to make decisions about their reproductive lives is central to their educational and economic achievement,^1^ their health^2^ and the health of their children. Women who are not able to plan their pregnancies are at increased risk for unsafe or repeat abortion.^2 3^ Mothers who are not able to time or space their pregnancies are more likely to experience obstetric fistula, uterine rupture and pregnancy-related mortality.^2 4^ Pregnancies fewer than 18 months apart are associated with increased risk of preterm birth,^5^ low birth weight and small for gestational age.^6^ When there is a reduction in time between births, children can face more competition with their siblings for parental attention, food, and access to education and medical care, especially in resource-limited settings.^7^

Intimate partner violence (IPV) is defined by the WHO as emotional, physical or sexual violence by a current or former partner.^8^ Recent meta-analyses suggest that IPV is associated with both decreased use of contraception^9^ and increased risk of unintended pregnancy and abortion.^10^ IPV may have both direct and indirect effects on a woman’s ability to space her pregnancies. Perpetrators of physical, sexual or emotional IPV may attempt to control their female partner’s fertility, affecting her ability to negotiate safe sexual practices. Reproductive coercion, also called fertility control, may co-occur with emotional, physical, or sexual IPV. Reproductive coercion can take the form of contraceptive sabotage; pregnancy pressure, unduly influencing a woman’s decision to become pregnant; and/or pregnancy coercion, unduly influencing the outcome of a pregnancy.^11 12^

In this manuscript, we assess whether IPV is associated with a reduction in the time between pregnancies. Prior to initiating these analyses, we reviewed existing literature on IPV and pregnancy spacing in low-and-middle-income countries (LMICs). Existing articles on pregnancy spacing in LMICs use cross-sectional data from the Demographic and Health Surveys (DHS) to estimate the association between IPV in the year prior to survey and the interval between live births.^13–15^ These articles exclude pregnancies that ended prior to term because of miscarriage or induced abortion or that did not result in live births, which likely underestimates the relation between IPV and pregnancy spacing given that IPV is associated with pregnancy termination.^10^ In this study we build on prior research by using monthly data from the DHS Reproductive Health (RH) Calendar and information on the year that IPV began to (1) temporally order both IPV and incident pregnancy and (2) include all pregnancies, whether or not those pregnancies resulted in live births. Accurate estimation of the effect of IPV on reproductive health is essential for quantifying the burden of disease associated with women’s experiences of violence.

## Methods

### Data

We used the most recent DHS data from all countries that asked women when IPV began in their most recent partnership as part of the Domestic Violence (DV) Module and that administered the RH Calendar, which collects monthly measures of women’s pregnancies, births and terminations, and contraceptive use over the 5 years prior to the survey. The DHS applies multistage, stratified probabilistic sampling to estimate important maternal and child health (MCH) indicators at the population level in most LMICs.^16^ Surveys were administered between 2005 and 2014 within each of the five DHS geographical regions. Some countries that administer the RH Calendar only collect calendar data from a subset of participants in the Women’s Household Survey. The DHS DV Module is administered to a subsample of women and girls aged 15–49 years included in the women’s survey (see table 1). DV Module participants signed an additional consent form and were only asked about their experience of violence if they could be interviewed without other adults present, in keeping with the WHO’s guidance for the protection of participants in partner violence research.^17^ Only ever-married or ever-partnered women and girls are asked about their experience of IPV. We restricted the data set to ever-partnered women aged 39 and younger at the time of survey because of age-specific differences in fertility intentions, and excluded women who reported that they were widowed at the time of survey.

**Table 1:** Regional and national distribution of women’s ever experience of any form of IPV (emotional, physical, or sexual)* across 29 low-income and middle-income countries surveyed as part of the DHS (n=95 159)

Country	Year	Sample size for women’s survey	Interviewed in Domestic Violence Module	Total women in analysis data set†	Total women in analysis data set who reported any IPV	
					n	%
Total		796 105	342 086	95 159	34 572	36.3
Central Asia		17 864	11 569	3563	1000	28.1
Kyrgyz Republic	2012	8208	6022	1808	569	31.5
Tajikistan	2012	9656	5547	1755	431	24.6
Latin America and Caribbean		100 166	104 199	17 824	6739	37.8
Colombia	2010	53 521	52 952	9004	3331	37.0
Honduras	2011	22 757	15 833	4443	1522	34.3
Peru	2012	23 888	35 414	4377	1886	43.1
North Africa, West Asia, Europe		44 487	20 908	5360	1485	27.7
Azerbaijan	2006	8444	5617	1299	274	21.1
Egypt	2014	21 762	6693	2607	816	31.3
Moldova	2005	7440	5695	1012	293	29.0
Ukraine	2007	6841	2903	442	102	23.1
South and South-East Asia		181 332	98 845	25 056	9390	37.5
Cambodia	2014	17 578	4307	1178	322	27.3
India	2005	124 385	83 703	19 906	7520	37.8
Nepal	2011	12 674	4197	1224	411	33.6
Pakistan	2012	13 558	3687	1609	672	41.8
Timor Leste	2009	13 137	2951	1139	465	40.8
Sub-Saharan Africa		226 128	106 565	43 356	15 958	36.8
Burkina Faso	2010	17 087	11 363	5444	913	16.8
Comoros	2012	5329	3341	1082	124	11.5
Ghana	2008	4916	2442	755	304	40.3
Kenya	2014	31 079	5657	2042	928	45.4
Malawi	2010	23 020	6229	2987	1137	38.1
Mali	2012	10 424	3459	1756	808	46.0
Mozambique	2011	13 745	6835	2789	1367	49.0
Namibia	2011	9176	2931	520	185	35.6
Nigeria	2013	38 948	27 634	11 035	3021	27.4
Rwanda	2005	11 321	4066	1768	671	38.0
Sierra Leone	2013	16 658	5185	1965	1026	52.2
Tanzania	2010	10 139	7047	2945	1319	44.8
Uganda	2011	8674	2056	945	577	61.1
Zambia	2013	16 441	11 778	5183	2589	50.0
Zimbabwe	2010	9171	6542	2140	989	46.2

*Sample restricted to ever-partnered women and girls who completed the Reproductive Health Calendar and who responded to any question related to physical, sexual or emotional IPV.

†Pakistan 2012 DHS did not measure sexual IPV; Colombia 2010 and Rwanda 2005 DHS did not measure emotional IPV.

DHS, Demographic and Health Surveys; IPV, intimate partner violence.

### Measures

Our time-varying exposure was IPV, measured using the DHS DV Module, which is based on the modified Conflict Tactics Scale^18^ as applied in the WHO Multi-Country Study on Women’s Health and Domestic Violence against Women. Women were asked for the year that their most recent partnership began; if they reported experiencing any form of IPV within that relationship, they were asked how many years they had been in the relationship before the IPV began. We estimated the hazard of any form of abuse (emotional, physical, or sexual) on incident pregnancy because the DHS did not collect data on when specific forms (emotional, physical, or sexual) of IPV started. We made the assumption that IPV continued in the relationship after the first reported instance. Close to 80% of women (79.6%) who reported that IPV began before the year prior to interview also reported experiencing IPV during the year prior to interview.

The main outcome was incident pregnancy. While the RH Calendar collects monthly data on pregnancy status for the last 5 years of a woman’s birth history, the DHS only records the intendedness of pregnancies that resulted in live births, rather than all incident pregnancies. For live-born children, the DHS asks, ‘at the time you became pregnant with (NAME), did you want to become pregnant then, did you want to wait until later, or did you not want to have any (more) children at all?’ We classified both mistimed and unwanted pregnancies as unintended pregnancies in our analysis of time-to-unintended pregnancy.

We created a directed acyclic graph to distinguish between variables that may be on the causal pathway between IPV and incident pregnancy, like contraception^9^ and parity,^10 19^ and probable confounders (see online supplementary figure S5). We adjusted for confounding by maternal age, marital status, maternal education, partner’s education, a country-specific measure of individual household wealth, described in detail elsewhere,^20^ and rural residence. We created a time-varying ratio of the number of surviving male children over the total number of surviving male and female children that included women’s full birth history and that changed with every live birth or infant or child death that occurred during the 5-year recall period. We excluded age at first cohabitation from our final models because of within-country collinearity. We considered restricted cubic splines with two knots for modelling continuous covariates: age and the proportion of surviving children who were male.

10.1136/bmjgh-2017-000304.supp3 Supplementary data

### Statistical analyses

We estimated the relation between IPV and time-to-pregnancy, measured in months. In addition, we estimated the association between IPV and time-to-unintended pregnancy where we restricted our inference to pregnancies that resulted in live births because the DHS only asked about the intendedness of pregnancies that resulted in live births. We defined the start of follow-up as the end of the first pregnancy (either miscarriage, termination or live birth) that began in the first 3 years of the 5-year birth history to allow time for the occurrence of the event of interest, incident pregnancy. Observations were censored at the end of the 5-year recall period or at the time of sterilisation of the respondent or her partner.

We used one-stage and two-stage meta-analyses to quantify and account for cross-country heterogeneity in the association between IPV and interpregnancy intervals. In the one-stage analysis, we applied Cox proportional hazards (PH)^21^ shared frailty models^22 23^ to estimate time-to-pregnancy and time-to-unintended pregnancy across countries, which allowed us to compare the survival curves by women’s experience of IPV. We used country-level frailties, the survival analysis equivalent of random effects in generalised mixed models,^24^ to account for unmeasured, country-level factors. We interacted confounders that violated the PH assumption with a restricted cubic spline of time with two knots to allow for a flexible relationship between non-proportional confounders and time.

For the two-stage meta-analysis, we applied stratified Cox PH models to estimate the relation between women’s experience of IPV and time-to-pregnancy or time-to-unintended pregnancy within each country and pooled country-specific HRs using random-effects meta-analysis. The two-stage analysis allowed us to explore country-level heterogeneity in the strength of the association between IPV and interpregnancy intervals. In contrast to the one-stage analysis, we stratified country-level models by confounders that violated the PH assumption, rather than interacting these covariates with time, to avoid making any inference about the relation between these confounders and time. We selected the country-specific model with the fewest stratifying variables and assessed whether the effect of IPV varied over time within each country. We addressed within-country collinearity issues by excluding one of the collinear variables. For the pooled estimate, country-level estimates were weighted by the inverse of the sum of the within-country sampling variance and the cross-country sampling variance.^25^ We used the I^2^ statistic to quantify the proportion of variation in effect estimates across studies due to actual variation rather than chance.^26^

In both the one- and the two-stage meta-analyses, when we found evidence that the effect of IPV varied over time, we compared different functional forms for time (linear, natural log, exponential) and included the form that resulted in the lowest value of the Akaike information criterion. We used Schoenfeld residuals to verify the PH assumption and included interactions between time and covariates that violated the PH assumption^27^ (see online supplementary figures S1 and S2 and supplementary tables S1 and S2 for the results from these model checks for the one- and two-stage meta-analyses, respectively).

10.1136/bmjgh-2017-000304.supp4 Supplementary data

In a sensitivity analysis, we included country-level proxy measures of women’s empowerment (the ratio of women to men in the workforce) and access to contraception (logged values of per capita gross domestic product (GDP) and healthcare expenditures, the percentage of the population in urban centres, and the prevalence of use of modern contraceptive methods) in the one-stage model of time-to-incident pregnancy to assess the degree to which these variables explained between-country heterogeneity. The ratio of women to men in the formal economy was not measured for Kenya or Nigeria, and these countries were excluded from the sensitivity analysis for the relation between IPV and time-to-incident pregnancy. Due to convergence issues, we only included the prevalence of modern contraception and logged GDP when exploring country-level heterogeneity in the relation between IPV and time-to-unintended pregnancy. Country-level markers of women’s empowerment and contraceptive access included a 2-year lag from the date of interview and were derived from the World Bank’s World Development Indicators Database.

The DHS imputes missing data on important MCH indicators.^28^ Only a small percentage of respondents who completed the RH Calendar and who answered any question about their experience of IPV were missing information on important confounders (n=1161; 1.2%), and these observations were excluded from the analyses. All analyses were conducted on Stata V.13.1.

## Results

### Characteristics of the sample

**Figure 1:**
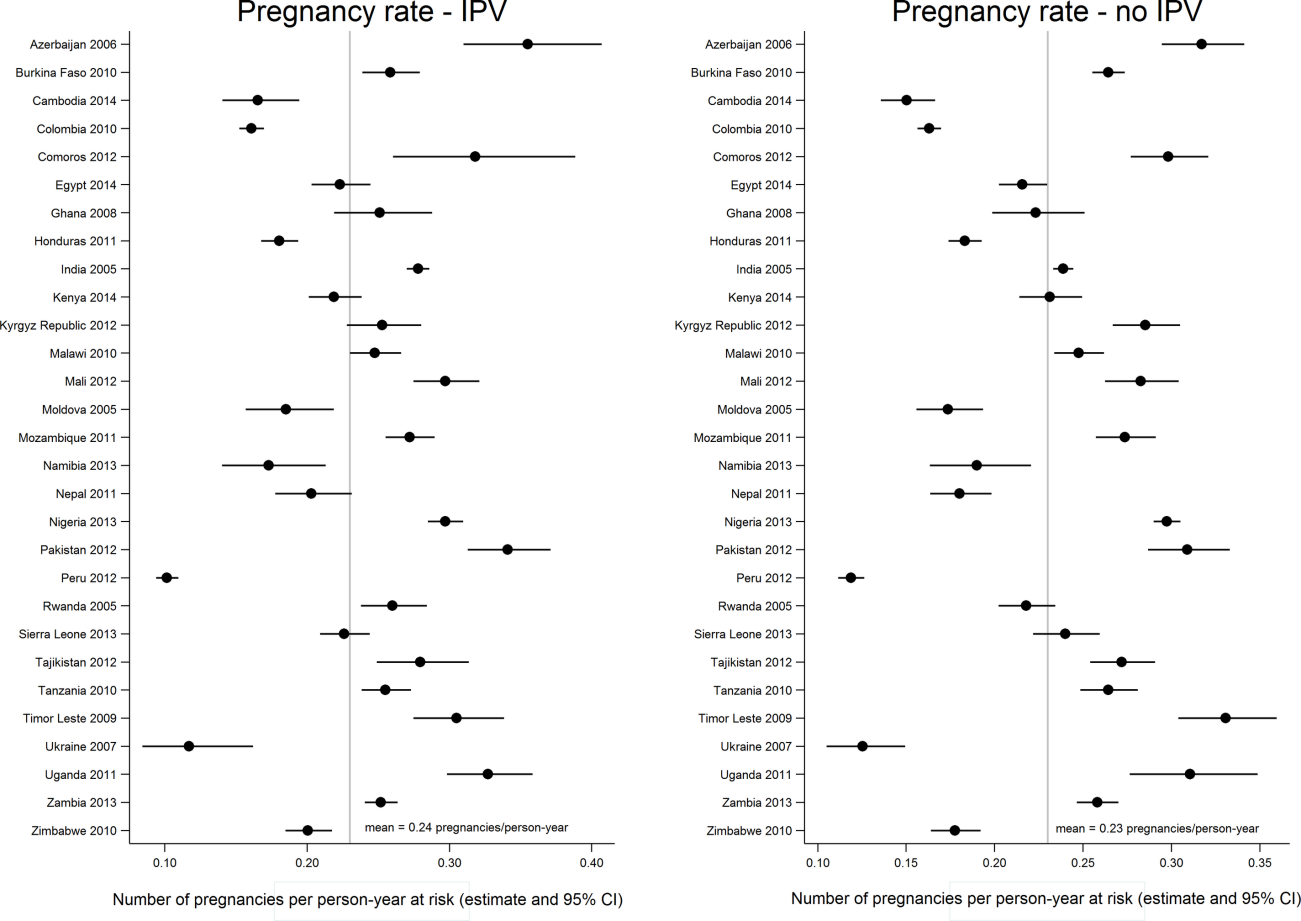
Distribution of the country-specific rates of incident pregnancy (pregnancies/person-years at risk) by ever experience of intimate partner violence (IPV). The figure presents the distribution of incident pregnancy rates across countries by whether women ever experienced any form of IPV (emotional, physical, or sexual) during follow-up. While there were some within-country differences in the pregnancy rates between women who did and who did not experience IPV, the mean pregnancy rates did not differ by experience of IPV.

Overall, 36% of women reported ever experiencing IPV (table 1). The percentage of women reporting emotional, physical, and/or sexual IPV was relatively consistent across regions, but varied widely between countries within each region. Women who participated in the Uganda 2011 and Sierra Leone 2013 surveys were the most likely to experience IPV (61% and 52%, respectively), while participants in the Comoros 2012 survey were the least likely to have experienced any form of IPV (12%). As illustrated in figure 1, the pregnancy rate varied widely across countries. For women who had never experienced IPV, the pregnancy rate ranged from 0.1 pregnancies/year in the Ukraine 2007 DHS to 0.3 pregnancies/year in the Timor Leste 2009 DHS. For women who experienced IPV, the pregnancy rate ranged from 0.1 pregnancies/year in the Peru 2012 DHS to 0.4 pregnancies/year in the Azerbaijan 2006 survey. The mean pregnancy rate was the same for women who had and had not experienced IPV (0.2 pregnancies/year), although the pregnancy rate varied by IPV status within some countries.

In table 2, we present respondents’ characteristics at the time of survey stratified by women’s ever experience of any form of IPV (physical, sexual or emotional). A smaller percentage of women who had ever experienced IPV reported completing secondary or higher levels of education (38% vs 44%). Similarly, women who experienced IPV were less likely to report that their partners had completed secondary or higher education (47% vs 51%). Compared with women who never experienced IPV, a lesser proportion of women who experienced IPV were married (74% vs 82%) and a greater proportion were divorced or separated (9% vs 3%). Compared with women who never experienced IPV, a higher proportion of women who experienced IPV had begun cohabitating with a partner prior to age 20 (73% vs 65%).

**Table 2:** Time-fixed characteristics of the analysis sample* measured at time of survey, stratified by women’s ever experience of any form of IPV (emotional, physical, or sexual; n=95 159)

	IPV		No IPV	
	n	%	n	%
Participant-level, time-fixed variables	34 572	36.3	60 587	63.7
Median age (min, max)	28.1	(15, 39)	28.2	(15, 39)
Median age at first cohabitation (min, max)	17.9	(5, 38)	18.6	(4, 38)
Age category				
15–19	1110	3.2	1772	2.9
20–24	8482	24.5	14 120	23.3
25–29	11 388	32.9	20 102	33.2
30–34	8423	24.4	15 341	25.3
35–39	5169	15.0	9252	15.3
Education level				
Higher than secondary/Secondary	12 979	37.5	26 681	44.0
Primary	11 758	34.0	16 119	26.6
No formal education	9831	28.4	17 779	29.3
Missing	4	0.0	8	0.0
Marital status				
Married	25 426	73.5	49 365	81.5
Living together	5922	17.1	9255	15.3
Divorced/not living together	3224	9.3	1967	3.2
Partner’s education level				
Higher than secondary/secondary	16 197	46.9	31 035	51.2
Primary	10 805	31.3	15 084	24.9
No formal education	7107	20.6	13 781	22.7
Missing	463	1.3	687	1.1
Age at first cohabitation				
Less than 20	25 143	72.7	39 398	65.0
20 or over	9429	27.3	21 189	35.0
Household-level variables				
Household wealth quintile				
Highest	4329	12.5	11 246	18.6
High	6529	18.9	11 641	19.2
Middle	7385	21.4	11 892	19.6
Low	8048	23.3	12 454	20.6
Lowest	8281	24.0	13 354	22.0
Rural residence				
Yes	21 618	62.5	37 859	62.5
No	12 954	37.5	22 728	37.5

*Sample restricted to ever-partnered women and girls who responded completed the Reproductive Health Calendar and who responded to any question related to physical, sexual or emotional IPV.

IPV, intimate partner violence.

### Time-to-pregnancy

The data set for the one-stage meta-analysis of the relation between IPV and time-to-pregnancy included 52 959 incident pregnancies from 90 446 participants in 29 countries, which represented 232 394 person-years at risk. The median interpregnancy interval was 29 months. We included a country-level frailty term to account for unmeasured, shared country-level factors related to pregnancy spacing. The estimated variance of the country-level frailty term was 0.09; the likelihood ratio test for the inclusion of the frailty term (H_0_ θ=0) was significant (P<0.0001), which suggests important cross-country heterogeneity in unmeasured determinants of time-to-pregnancy. Including country-level markers of women’s inclusion in the workforce and contraceptive access decreased the variance of the country-level frailty term from 0.09 to 0.04, but the frailty term was still statistically significant, which indicates residual cross-country heterogeneity. We found evidence that the effect of IPV on the hazard of incident pregnancy decreased over time and included an interaction term between IPV and (log) time in the one-stage meta-analysis (see online supplementary table S3 for a comparison of model fits using different specifications of time for the IPV-time dependency). We modelled age as a restricted cubic spline with two knots. We did not find evidence that modelling the proportion of surviving male children using restricted cubic splines contributed to model fit and modelled the proportion of surviving male children as a continuous, linear variable. We included an interaction between a restricted cubic spline of time with two knots and age, marital status, maternal and partner’s education level, household wealth quintile, and urban residence because the effects of these covariates were not constant over time.

**Figure 2:**
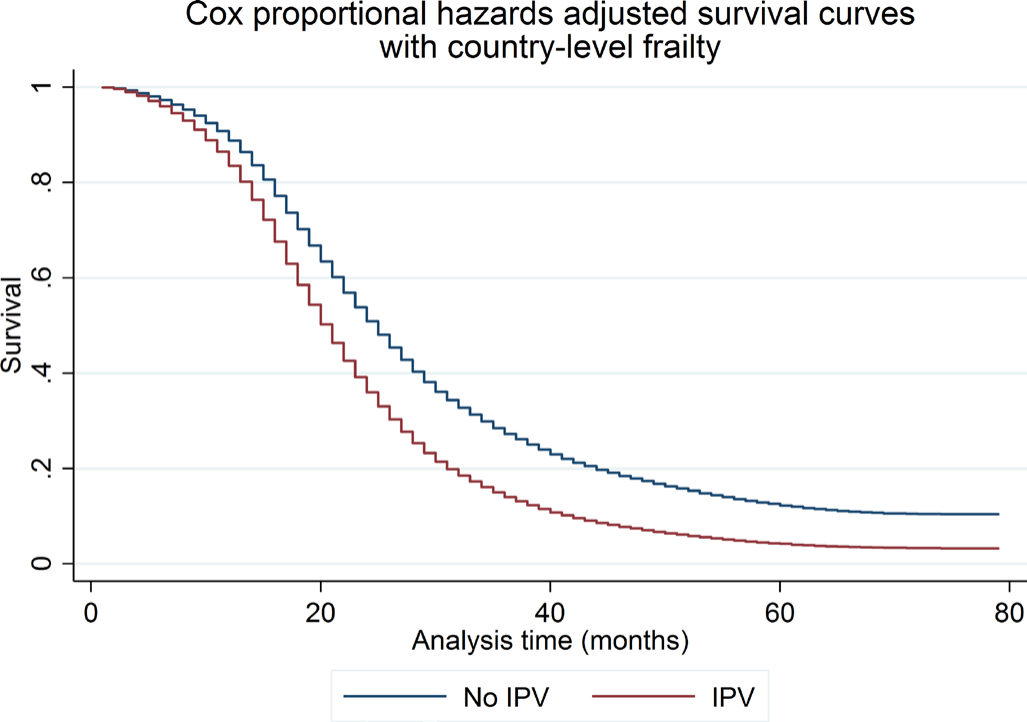
One-stage meta-analysis of intimate partner violence (IPV) and time-to-incident pregnancy modelled using shared frailty Cox proportional hazards. The graph presents Cox proportional hazards shared frailty survival curves for the one-stage meta-analysis of the relation between IPV and time-to-incident pregnancy. The effect of IPV was not constant over time and the shared frailty model includes an interaction between IPV and log time, which allows the effect of IPV to diminish over time. The Cox proportional hazards model is adjusted for the proportion of surviving children who were male and includes interaction terms between a restricted cubic spline of time with two knots and confounders that were not proportional over time, including maternal age (modelled as a restricted cubic spline with two knots), marital status, maternal education, partner’s education, household wealth and rural residence. The model is conditional on the country-level frailty terms that account for unobserved, country-level factors that affect the relation between IPV and interpregnancy intervals.

In figure 2, we present the adjusted survival curves for the shared frailty Cox PH models. The difference between the survival curves suggests that women who experienced IPV had a higher rate of incident pregnancy than women who did not, although that difference decreases over time. In table 3, we present results from the one-stage meta-analysis. The proportion of surviving male children was associated with increased time between pregnancies (HR: 0.93; 95% CI 0.91 to 0.95), which implies that women who had a higher percentage of surviving male children were more likely to wait to have another pregnancy. After adjusting for maternal age, marital status, maternal and partner’s education, proportion of surviving male children, household wealth quintile, and rural residence, the likelihood of incident pregnancy was 51% higher for women who experienced any form of IPV (emotional, physical, sexual) during follow-up than for women who did not (95% CI 1.38 to 1.66). The interaction between IPV and (log) time suggests that the effect of exposure to IPV on pregnancy spacing decreases over time.

**Table 3:** Cox PH shared frailty models for the association between IPV and time-to-pregnancy for all incident pregnancies (n=90 446 women; 52 959 pregnancies; 232 394 person-years), and for IPV and time-to-unintended pregnancy for pregnancies that resulted in live births (n=92 848 women; 13 541 unintended pregnancies; 310 319 person-years)

	Shared frailty Cox PH for all incident pregnancies*	Shared frailty Cox PH for unintended pregnancies that resulted in live births*
Participant-level, time-varying variables	HR (95% CI)	HR (95% CI)
Intimate partner violence†	1.51 (1.38 to 1.66)	1.30 (1.25 to 1.34)
Intimate partner violence × ln(time)‡	0.89 (0.86 to 0.92)	
Number of surviving boys/total surviving children	0.93 (0.91 to 0.95)	0.99 (0.95 to 1.03)
Variance of the country-level frailty (SE)	0.09 (0.02)	0.34 (0.09)

*Includes an interaction between a restricted cubic spline with two knots for time and age (modelled as a restricted cubic spline with two knots), marital status, maternal education, partner’s education, household wealth quintile and rural residence, conditional on country-level frailty terms.

†Includes emotional, physical and/or sexual violence.

‡IPV-time dependency not included in Cox PH models for time-to-unintended pregnancy.

IPV, intimate partner violence; PH, proportional hazards.

Because we were specifically interested in the relation between IPV and inadequate spacing, we estimated survival curves for the 18 months following the index pregnancy (see online supplementary figure S3). When limiting inference to the 18 months following the index pregnancy, the results were comparable with those from the analysis of all pregnancies; women who experienced IPV were 41% more likely to become pregnant within the 18 months after the index pregnancy than women who did not experience IPV during that time (95% CI 1.21 to 1.64).

10.1136/bmjgh-2017-000304.supp2 Supplementary data

**Figure 3:**
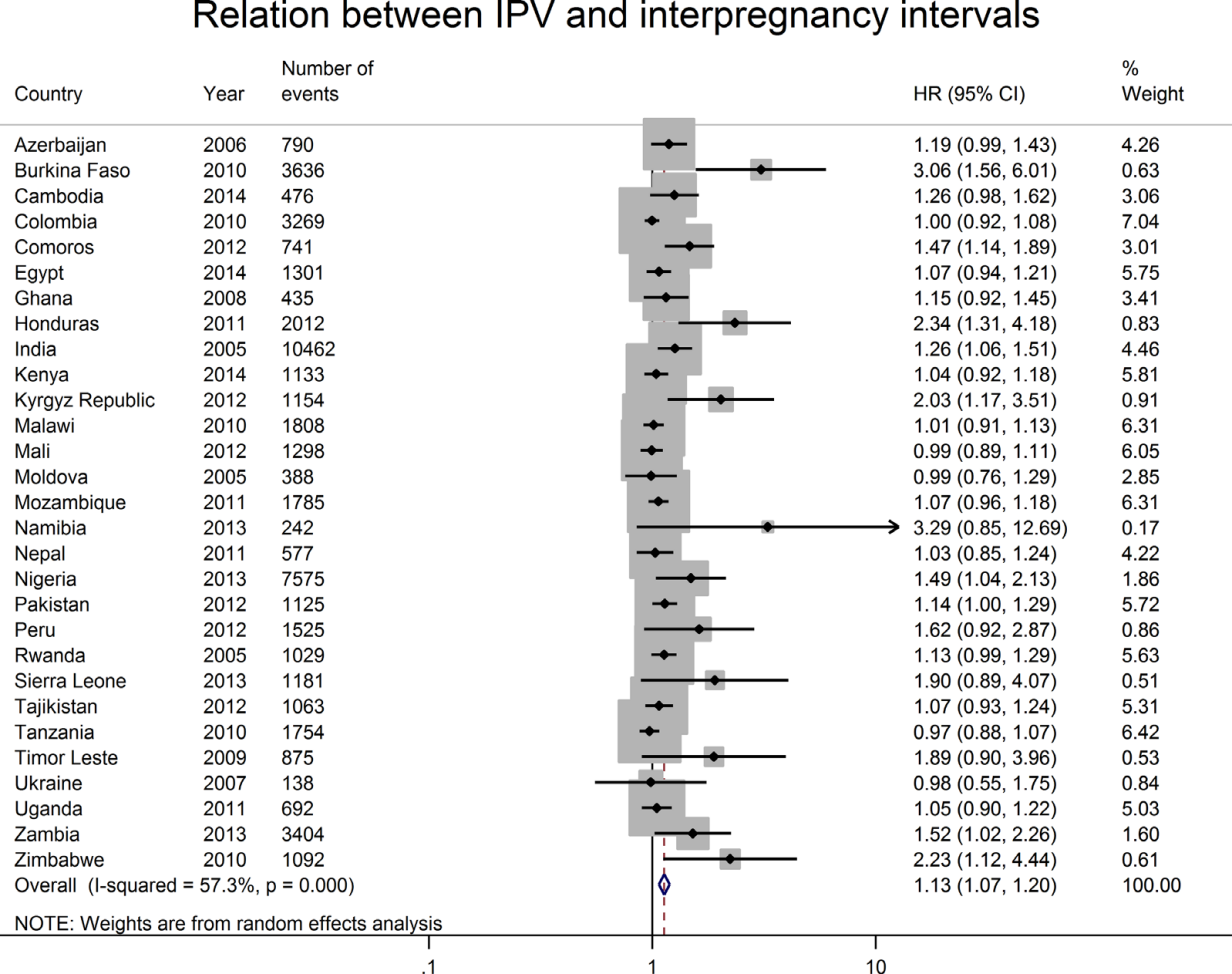
Two-stage random-effects meta-analysis of country-specific measures of the association between intimate partner violence (IPV) and time-to-incident pregnancy. The figure presents the forest plot for the relation between IPV and time-to-incident pregnancy. We stratified country-level HRs by confounders that were not constant over time to avoid inferring the shape of the relation between the confounders and time. We selected the country-level estimate with the fewest number of strata and addressed issues of country-level collinearity by excluding one of the collinear variables. We included an interaction term between IPV and time if there was evidence that the relation between IPV and interpregnancy intervals varied over time for a given country. Country-level estimates were weighted by the inverse of the sum of the within-country sampling variance and the cross-country sampling variance. The shaded area around the point estimate reflects the weight given to each country’s estimate in the pooled estimate.

To better understand cross-country heterogeneity, we used Cox PH models to estimate the relation between women’s experience of IPV and time-to-incident pregnancy within each country. In online supplementary table S1, we present effect estimates for the final model selected for each country and used in the two-stage meta-analysis of the relation between IPV and time-to-pregnancy. The stratifying variables and whether or not the effect of IPV diminished over time differed across countries. In figure 3, we present a forest plot of the country-level estimates for the relation between IPV and incident pregnancy. We found that IPV had the strongest effect on reducing the time between pregnancies in Burkina Faso (HR: 3.06; 95% CI 1.56 to 6.01) and Honduras (HR: 2.34; 95% CI 1.31 to 4.18). All estimates either included the null value or suggested that IPV was associated with a reduction in the time between pregnancies. The I^2^ statistic of 57 (95% CI 35 to 72) indicates a moderate-to-high level of heterogeneity in the meta-analytic estimate.^29^ The pooled estimate from the two-stage random-effects meta-analysis was slightly lower than that of the one-stage analysis (HR: 1.13, 95% CI 1.07 to 1.20 vs HR: 1.51, 95% CI 1.38 to 1.66 in the one-stage analysis), although the effect of IPV on interpregnancy intervals diminishes over time in the one-stage model.

### Time-to-unintended pregnancy

**Figure 4:**
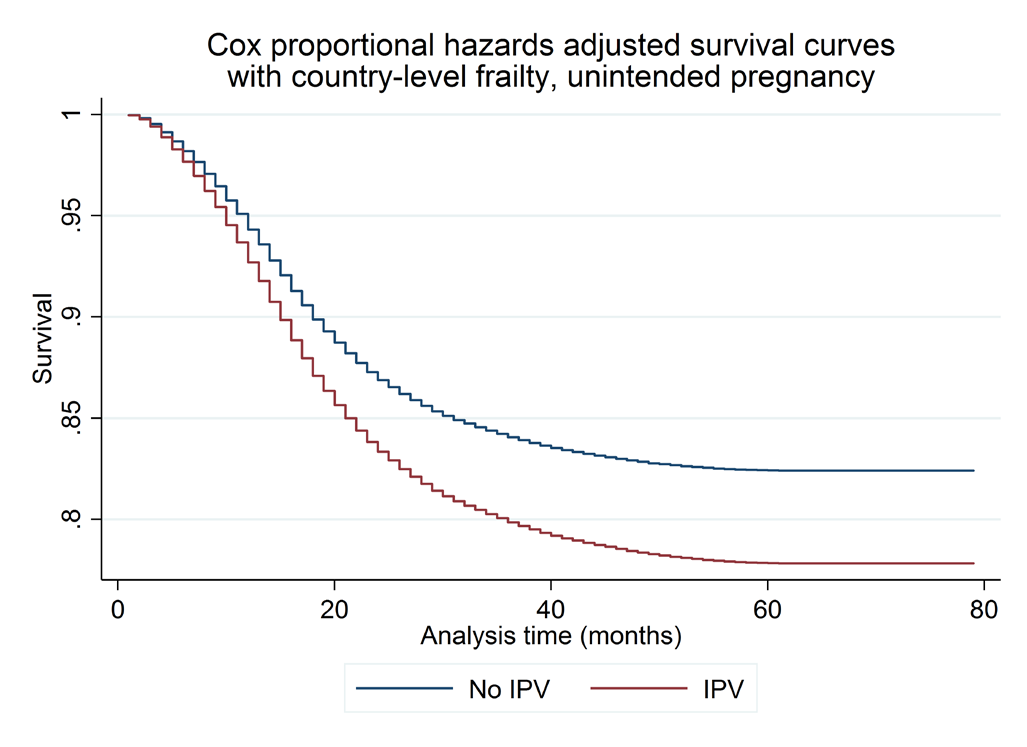
One-stage meta-analysis of intimate partner violence (IPV) and time-to-unintended pregnancy using shared frailty Cox proportional hazards. The model presents Cox proportional hazards shared frailty survival curves for the one-stage meta-analysis of the relation between IPV and time-to-unintended pregnancy, which is limited to pregnancies that resulted in live births. The Cox proportional hazards model is adjusted for the proportion of surviving children who were male and includes interaction terms between a restricted cubic spline of time with two knots and confounders that were not proportional over time, including maternal age (modelled as a restricted cubic spline with two knots), marital status, maternal education, partner’s education, household wealth, and rural residence. The model is conditional on country-level frailty terms that account for unobserved, country-level factors that affect the relation between IPV and time-to-unintended pregnancy.

In figure 4, we present the adjusted survival curves for the one-stage meta-analysis of time-to-unintended pregnancy, which is limited to pregnancies that resulted in live births because the DHS only asked about the intendedness of live-born children. The median time-to-unintended pregnancy was 43 months. The variance of the country-level frailty term was 0.34; the likelihood ratio test for the inclusion of the frailty term (H_0_ θ=0) was significant (P<0.0001), which suggests important cross-country heterogeneity in unmeasured determinants of time-to-unintended pregnancy. The inclusion of country-level markers of contraceptive access (GDP and prevalence of modern contraceptive use) did not decrease the variance of the frailty term. In contrast to the analysis of all incident pregnancies, we did not find evidence that the association between IPV and time-to-unintended pregnancy varied over time. As with the analysis of all pregnancies, we modelled age as a restricted cubic spline with two knots and the proportion of surviving male children as a linear variable. We included an interaction between a restricted cubic spline of time with two knots and age, marital status, maternal and partner’s education level, household wealth quintile, and urban residence to account for variation in these effects over time. As illustrated in figure 4, women who experienced IPV had a higher rate of unintended pregnancy than women who did not. We present results for the one-stage meta-analysis of time-to-unintended pregnancy in table 3. In contrast to results from the analysis of all pregnancies, whether or not they resulted in a live birth, the proportion of surviving male children was not related to time-to-unintended pregnancy (HR: 0.99; 95% CI 0.95 to 1.03). In the adjusted model, women’s experience of any form of IPV (emotional, physical or sexual) was associated with a 30% increase in the likelihood of unintended pregnancy (95% CI 1.25 to 1.34; n=13 541 pregnancies, 92 848 women, 310 319 person-years at risk). We present a comparison of survival curves for women who did and did not experience IPV for the 18 months following the index pregnancy in online supplementary figure S4. Compared with women who did not experience IPV during follow-up, women who experienced IPV were 35% more likely to have an unintended pregnancy within 18 months of the index pregnancy (95% CI 1.28 to 1.43).

**Figure 5:**
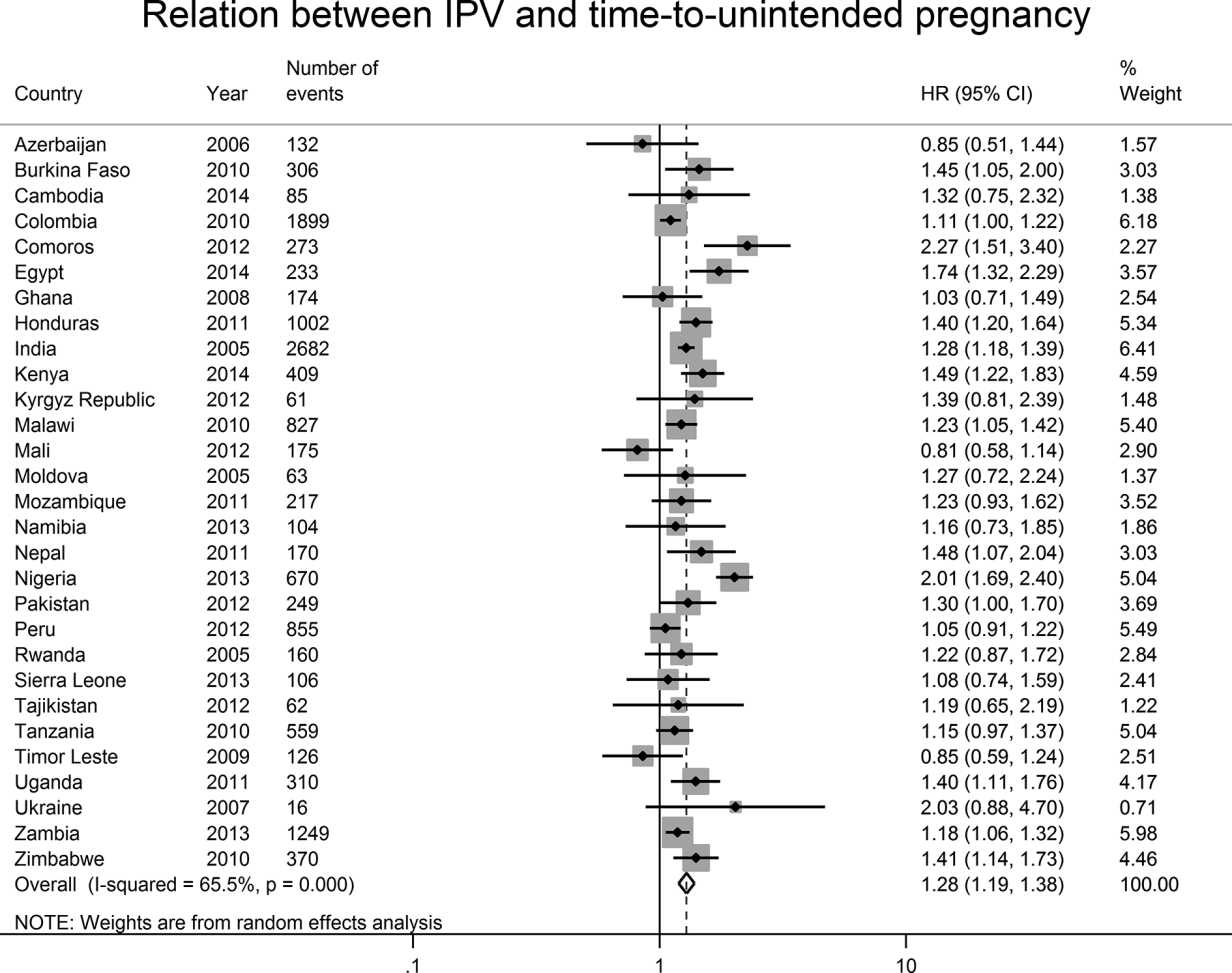
Two-stage random-effects meta-analysis of country-specific measures of association between intimate partner violence (IPV) and time-to-unintended pregnancy. The figure presents the forest plot for the relation between IPV and time-to-unintended pregnancy, which is limited to pregnancies that resulted in live births. We stratified country-level HRs by confounders that were not constant over time to avoid making inference about the shape of the relation between these confounders and time. We selected the country-level estimate with the fewest number of strata and addressed issues of country-level collinearity by excluding one of the collinear variables. We found no evidence that the interaction between IPV and time-to-unintended pregnancy varied over time within any country. Country-level estimates are weighted by the inverse of the sum of the within-country sampling variance and the cross-country sampling variance. The shaded area around the point estimate reflects the weight given to that country in the pooled estimate.

For the two-stage meta-analysis, we applied country-specific Cox PH models to estimate the relative time-to-unintended pregnancy for women who were and who were not exposed to IPV. In keeping with the two-stage meta-analysis of time-to-incident pregnancy, in the two-stage meta-analysis of time-to-unintended pregnancy, the stratifying variables varied widely across countries. We did not find evidence that the effect of IPV varied by time in any of the 29 countries. In online supplementary table S2, we present the final model for each country, including the stratifying variables, and which variables were dropped because of within-country collinearity. In figure 5, we present a forest plot of the country-level point estimates for the association between IPV and time-to-unintended pregnancy. We found that IPV was associated with the highest risk of unintended pregnancy in Comoros (HR: 2.27; 95% CI 1.51 to 3.40) and Nigeria (HR: 2.01; 95% CI 1.69 to 2.40). Point estimates either included the null value or indicated that IPV was associated with a decrease in the time-to-unintended pregnancy. We used random-effects meta-analysis to estimate the pooled HR. The I^2^ statistic of 66 (95% CI 49 to 77) indicates a moderate-to-high level of heterogeneity in the meta-analytic estimate.^25^ The estimate from the one-stage meta-analysis was similar to the pooled estimate from the two-stage meta-analysis (HR: 1.30, 95% CI 1.25 to 1.34; and HR: 1.28, 95% CI 1.19, to1.38, respectively).

## Discussion

About a third (36%) of the women in the analysis data set had ever experienced emotional, physical, and/or sexual IPV. For the one-stage analysis, considering 52 959 incident pregnancies, which represented 232 394 person-years at risk, women’s experience of IPV was associated with a 51% increase in the likelihood of incident pregnancy, although the effect of IPV diminished over time. When limiting our inference to unwanted pregnancies that resulted in live births, women’s experience of IPV was associated with a 26% increase in the likelihood of unintended pregnancy (n=13 545 pregnancies, 92 858 women, 310 349 person-years at risk). In the two-stage model, women’s experience of IPV was associated with a 13% increase in the probability of incident pregnancy and with a 28% increase in the likelihood of unintended pregnancy.

We found that the effects of predictors on time to pregnancy were not constant across countries, which suggests that models that assume homogeneity of effects across countries may lead to biased estimates. While the association between different covariates and interpregnancy intervals differed across countries, the association between IPV and interpregnancy intervals was relatively constant. In the two-stage meta-analyses, country-level estimates of time-to-incident pregnancy and time-to-unintended pregnancy either included the null value or indicated that IPV was associated with shorter interpregnancy intervals.

In the one-stage models, the magnitude of the relation between IPV and interpregnancy intervals decreased over time in the analysis of all incident pregnancies but not in the analysis of unintended pregnancies that resulted in live births. This difference is likely related to unintended pregnancy. Pregnancy intendedness was only reported for pregnancies that resulted in live births; therefore, the analysis of all pregnancies includes both unintended and intended pregnancies. Pregnancies that occur in short succession are more likely to have been unintended so the magnitude of the relation between IPV and time-to-all incident pregnancies is strongest in the 9 months following the end of the pregnancy that begins the interpregnancy interval (ie, when the probability of an unintended pregnancy is the highest), but the magnitude of the relation between IPV and time-to-unintended pregnancy is constant over time. We present period-specific HRs for time-to-all incident pregnancies and time-to-unintended pregnancy in online supplementary table S4 to further illustrate the role of pregnancy intendedness in the relation between IPV and interpregnancy intervals.

10.1136/bmjgh-2017-000304.supp5 Supplementary data

The variance of the frailty terms in the one-stage analyses and the I^2^ statistic in the two-stage analyses suggests a high level of residual between-country heterogeneity, even after the inclusion of country-level markers of women’s empowerment and contraceptive access in the one-stage models. The high level of heterogeneity across countries is not surprising given that a number of determinants of pregnancy spacing (eg, ideal family size, access to induced abortion) were not included in this analysis. In addition, determinants of pregnancy spacing, like breast feeding, contraceptive use, norms around ideal family size, exposure to conflict settings and displacement, and access to induced abortion, vary significantly by country. When interpreting the findings from the one-stage analysis, the reader should focus on the survival curves rather than point estimates because the average HR can obscure important changes in survival over time.^30^ When interpreting findings from the two-stage analysis, the reader should keep in mind the limitations of the average HR and focus on the country-specific estimates rather than the meta-analytic estimate because of the high level of heterogeneity in the meta-analytic estimates.

Findings from this study are in keeping with those of a smaller longitudinal study based in the USA that found that IPV was associated with reduced time between pregnancies,^31^ and with findings from cross-sectional studies that applied DHS data including a study of the association between IPV and unintended pregnancies that resulted in live births that used the Colombia 2000 DHS^13^ and from a multilevel study by Hung *et al* 14 that estimated the association between IPV and birth spacing in a number of countries in Sub-Saharan Africa. The Hung *et al* manuscript included random effects at the woman, village and country levels and found that both individual history of physical or sexual IPV and village-level prevalence of IPV were associated with shortened intervals between live births. In the one-stage analysis of all incident pregnancies, we found that women with more sons were slightly more likely to wait to have another birth. While there is an extensive, often conflicting, literature on son preference,^32 33^ we are not aware of recent research into the association between the proportion of surviving children who were male and pregnancy spacing in the LMICs included in this analysis.

### Strengths and limitations

This analysis has a number of strengths. We used one-stage meta-analysis to assess the shape of the relation between IPV and interpregnancy intervals and two-stage meta-analysis to explore cross-country variation in the magnitude of the relation between IPV and interpregnancy intervals. This is the largest multicountry analysis of the relation between IPV and interpregnancy intervals, and to our knowledge the only study to estimate the relation between IPV and all incident pregnancies, whether or not they resulted in live births, at the population level for all of the LMICs included in this analysis. While most literature on the intersection of IPV and pregnancy spacing uses the interval between live births,^34^ given the association between IPV and repeat abortion,^10^ interpregnancy intervals may offer a more accurate estimate of the association between IPV and women’s ability to space and limit their pregnancies. The rich data recorded in the RH Calendar are often not used because of the coding required to transform the data into the appropriate format. We include the code needed to transform RH Calendar data from a character string to an event data file that can be used for survival analysis in online supplementary text S1.

This analysis has several limitations. As mentioned previously, we made the assumption that women continued to experience IPV after the year that IPV began within their relationship. While we evaluated that assumption by examining the proportion of women who reported experiencing IPV both prior to the year prior to interview and during the year prior to interview, ideally, we would have had time-varying information on women’s exposure to IPV and on important confounders, like education and household wealth. In addition, we only had data on the year, rather than on the month that IPV began. Different forms of IPV may be only somewhat correlated^35^ and may have different effects on pregnancy spacing. In this analysis, we were not able to isolate the effects of the different forms of IPV measured by the DHS (emotional, physical, and sexual) and had to limit our inference to the relation between any form of IPV and interpregnancy intervals.

The duration of breast feeding,^36^ parity and contraceptive use are important predictors of pregnancy spacing. We were not able to include breast feeding in our models because, in the vast majority of countries, the duration of breast feeding was only measured for respondents’ most recent pregnancy. The reduced interval between incident pregnancies may be caused by an increase in induced abortions or by a reduction in women’s ability to negotiate contraception in the context of IPV. Prior studies have shown that IPV affects contraceptive use^9^ and parity^10 19^; contraceptive use is known to affect pregnancy status and parity is a strong predictor of pregnancy spacing. We estimated the total association between IPV and incident pregnancy, rather than examining the role of contraception or of parity as mediators of the relation between IPV and pregnancy given that Cox PH models cannot be used to assess mediation with frequently occurring outcomes,^37^ as is incident pregnancy in this data set. To better understand the causal relation between IPV and pregnancy spacing, future analyses could assess the direct effect of IPV on unwanted or mistimed pregnancy and the indirect effects mediated by contraception and induced abortion. Future research that quantifies the relative contribution of these pathways to shorter interpregnancy intervals could be used to inform interventions designed to help women space and limit their pregnancies.

Lastly, this analysis uses women’s self-report of the timing of their pregnancies in the 5 years prior to interview. While interviewers use cues to improve the accuracy of these self-reports,^38^ as with any study that involves recall, this study is subject to bias. A study in Bangladesh found that women who have experienced the highest numbers of pregnancies over the recall period may be least likely to accurately report those pregnancies.^38^ Despite this limitation, the DHS RH Calendar is the only source of population-level, longitudinal data on women’s incident pregnancies for most of the LMICs included in this analysis.

### Implications for practice

While findings from this analysis can help to quantify the burden of disease associated with women’s experience of IPV, researchers need to understand how IPV modifies pregnancy spacing to develop targeted, evidence-based interventions. The relation between IPV and pregnancy spacing both affects and is affected by women’s empowerment, individual and community beliefs about the acceptability of partner violence, gender inequities and poverty, and operates within the context of structural and cultural determinants of family size, breast feeding and contraceptive use. Interventions designed to help women who experience violence space their pregnancies will need to appropriately account for differences in the relative importance of these factors across and within countries. Current WHO guidelines specify that providers should ask women about their exposure to IPV when assessing RH conditions that may be caused or complicated by IPV.^8^ The American College of Obstetricians and Gynecologists provides comprehensive guidance for providers to ask women about their experience of reproductive coercion.^39^ Insights on how IPV affects interpregnancy intervals gained from regular clinical practice may help to inform the design of interventions to help women who experience violence space their pregnancies. Recent clinical guidance suggests that providers caring for women who experience reproductive coercion should offer contraceptive methods that are less dependent on negotiation or less susceptible to partner sabotage (eg, intrauterine device and implant) while counselling women about pregnancy spacing and safety planning.^40^

## Conclusion

Women’s experience of emotional, physical, or sexual IPV is associated with decreased time between pregnancies and an increased rate of unintended pregnancy. Pregnancy and birth spacing are closely associated with maternal death and disability and are important predictors of both short-term and long-term MCH, economic, and educational outcomes. Quantifying the relation between IPV and women’s RH outcomes is an emerging field in gender-based violence research and an important part of understanding the burden of disease related to IPV.^41^ Because of the centrality of pregnancy spacing to MCH, results from this analysis may help inform estimates of the burden of disease related to women’s experience of IPV.

10.1136/bmjgh-2017-000304.supp1 Supplementary data
