# Supplementary material for: Intimate partner violence and pregnancy spacing: results from a meta-analysis of individual participant time-to-event data from 29 low-and-middle-income countries
Source: BMJ Glob Health. 2018 Jan 13;3(1):e000304. doi: 10.1136/bmjgh-2017-000304 (PMC5859805; doi:10.1136/bmjgh-2017-000304)
Supplement: Supplementary data [file bmjgh-2017-000304supp003.pdf]

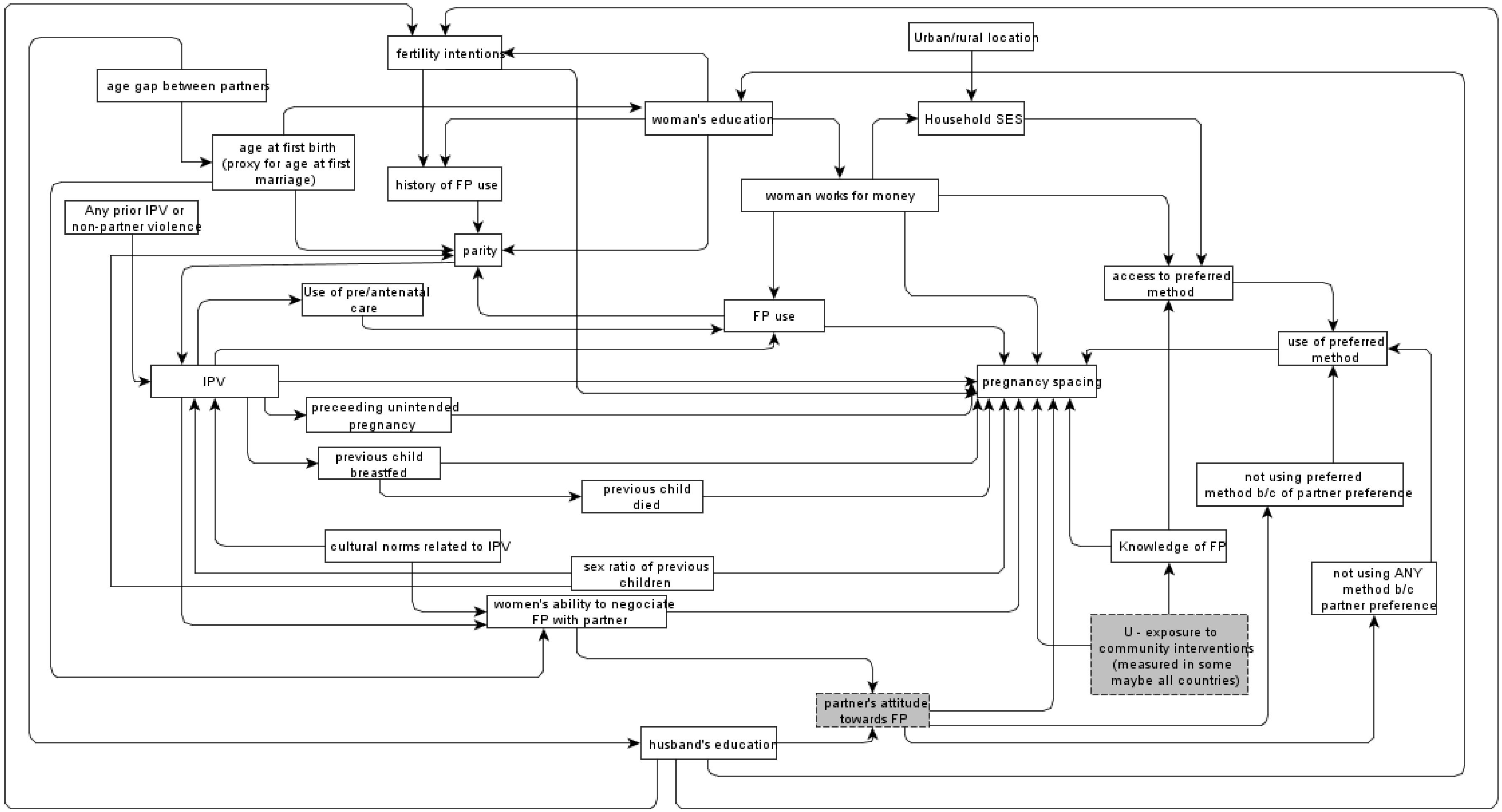

Figure S5. Directed acyclic graph representing the hypothesized causal relation between women's experience of IPV and pregnancy spacing.
