# Supplementary material for: Intimate partner violence and pregnancy spacing: results from a meta-analysis of individual participant time-to-event data from 29 low-and-middle-income countries
Source: BMJ Glob Health. 2018 Jan 13;3(1):e000304. doi: 10.1136/bmjgh-2017-000304 (PMC5859805; doi:10.1136/bmjgh-2017-000304)
Supplement: Supplementary data [file bmjgh-2017-000304supp004.pdf]

Table S1. Country-level stratified Cox proportional hazards models of time-to-incident pregnancy

	Azerbaijan 2006			Burkina Faso 2010			Cambodia 2014			Colombia 2010		
	Beta	SE	P-value	Beta	SE	P-value	Beta	SE	P-value	Beta	SE	P-value
**IPV**	0.173	0.095	0.069	1.120	0.344	0.001	0.229	0.128	0.075	-0.00235	0.039	0.952
**Age**												
15-19	Ref											
20-24	-0.120	0.527	0.820									
25-29	-0.607	0.526	0.248									
30-34	-1.130	0.532	0.034									
35-39	-1.214	0.536	0.024									
**Maternal education**												
no education	Ref			Ref								
primary	-0.186	0.338	0.582	-0.119	0.056	0.032						
secondary/higher	-0.444	0.211	0.035	-0.170	0.099	0.087						
**Marital status**												
married	Ref						Ref					
living together	0.593	0.258	0.021				-0.0813	0.698	0.907			
divorced/not living together	-1.134	0.392	0.004				-0.530	0.325	0.103			
**Partner's education**												
no education	Ref			Ref			Ref			Ref		
primary	-0.0516	0.687	0.940	0.00979	0.054	0.856	-0.263	0.175	0.133	-0.133	0.081	0.098
secondary or higher	0.449	0.570	0.430	-0.295	0.085	0.000	-0.190	0.184	0.302	-0.119	0.083	0.151
**Age first cohabitation**												
<20	Ref			Ref			Ref					
>=20	0.291	0.083	0.000	0.0963	0.049	0.048	0.475	0.106	0.000			
**Minority**	-0.144	0.214	0.501	0.0616	0.034	0.067				0.350	0.164	0.032
**Wealth quintile**												
Lowest	Ref											
Low	-0.110	0.103	0.285									
Middle	-0.103	0.113	0.362									
High	-0.233	0.127	0.067									
Highest	-0.192	0.146	0.188									
**Rural residence**	-0.0984	0.093	0.288	0.157	0.053	0.003	-0.354	0.151	0.019	-0.110	0.048	0.021
**Proportion of boys/all surviving**	-0.163	0.085	0.055	0.00663	0.044	0.880	0.0551	0.115	0.633	-0.0256	0.043	0.548
**IPVXlntime**				-0.330	0.106	0.002						
Observations	29917			170929			39274			254303		

	Azerbaijan 2006			Burkina Faso 2010			Cambodia 2014			Colombia 2010		
	Beta	SE	P-value	Beta	SE	P-value	Beta	SE	P-value	Beta	SE	P-value
PH test P-value	0.266			0.418			0.304			0.059		
Stratified by					Age, marital status, wealth quintile			Age, maternal education, wealth quintile			Age, marital status, maternal education, wealth quintile	
Dropped because of collinearity								Minority			Age at first cohabitation	

	Comoros 2012			Egypt 2014			Ghana 2008			Honduras 2011		
	Beta	SE	P-value	Beta	SE	P-value	Beta	SE	P-value	Beta	SE	P-value
**IPV**	0.384	0.130	0.003	0.0687	0.064	0.285	0.143	0.117	0.222	0.851	0.296	0.004
**Age**												
15-19	Ref						Ref					
20-24	-0.215	0.209	0.305				0.498	0.423	0.239			
25-29	-0.364	0.205	0.075				0.218	0.419	0.603			
30-34	-0.613	0.212	0.004				0.00791	0.424	0.985			
35-39	-0.747	0.216	0.001				-0.424	0.431	0.326			
**Maternal education**												
no education	Ref			Ref			Ref			Ref		
primary	0.0750	0.093	0.420	-0.0625	0.126	0.619	0.183	0.133	0.168	-0.154	0.089	0.083
secondary/higher	-0.0802	0.103	0.436	-0.0105	0.088	0.905	-0.173	0.142	0.223	-0.307	0.108	0.004
**Marital status**												
married	Ref			Ref			Ref					
living together	0.124	0.327	0.704				-0.166	0.130	0.202			
divorced/not living together	-0.487	0.207	0.019	-3.200	1.028	0.002	-0.827	0.302	0.006			
**Partner ‘s education**												
no education	Ref			Ref			Ref			Ref		
primary	-0.157	0.097	0.104	0.168	0.125	0.177	0.0136	0.182	0.941	-0.163	0.077	0.033
secondary or higher	-0.0739	0.093	0.426	0.300	0.101	0.003	-0.0330	0.147	0.822	-0.266	0.098	0.007
**Age first cohabitation**												
<20	Ref									Ref		
>=20	0.245	0.083	0.003							0.134	0.057	0.019
**Minority**	0.00456	0.368	0.990				-0.0223	0.166	0.893	0.0441	0.142	0.756
**Wealth quintile**												
Lowest	Ref			Ref			Ref					
Low	-0.112	0.106	0.290	-0.152	0.092	0.100	0.121	0.131	0.357			
Middle	-0.186	0.109	0.089	-0.224	0.093	0.016	-0.142	0.165	0.390			
High	-0.433	0.129	0.001	-0.177	0.106	0.095	-0.120	0.185	0.515			

	Comoros 2012			Egypt 2014			Ghana 2008			Honduras 2011		
	Beta	SE	P-value	Beta	SE	P-value	Beta	SE	P-value	Beta	SE	P-value
Highest	-0.105	0.141	0.457	-0.298	0.127	0.019	-0.0319	0.237	0.893			
**Rural residence**	0.134	0.083	0.108	-0.0412	0.088	0.641	0.137	0.145	0.342	-0.126	0.065	0.054
**Proportion of boys/all surviving**	0.0192	0.105	0.855	-0.333	0.073	0.000	-0.0965	0.109	0.378	-0.0412	0.053	0.439
**IPVXlntime**										-0.243	0.093	0.009
Observations	29742			76264			22639			140343		
PH test P-value	0.985			0.713			0.161			0.405		
Stratified by					Age						Age category, marital status, wealth quintile	
Dropped because of collinearity					Minority, age first cohabitation			Age first cohabitation				

	India 2005			Kenya 2014			Kyrgyz Republic 2012			Malawi 2010		
	Beta	SE	P-value	Beta	SE	P-value	Beta	SE	P-value	Beta	SE	P-value
**IPV**	0.235	0.091	0.010	0.0406	0.063	0.519	0.707	0.281	0.012	0.0145	0.054	0.787
**IPVXlntime**	-0.0511	0.030	0.087				-0.247	0.094	0.009			
**Partner's education**												
no education	Ref			Ref						Ref		
Primary	-0.0206	0.032	0.515	-0.0792	0.102	0.437				-0.0273	0.080	0.735
secondary or higher	-0.0169	0.027	0.534	-0.267	0.119	0.024				-0.219	0.095	0.022
**Age first cohabitation**												
<20	Ref			Ref			Ref			Ref		
>=20	0.235	0.027	0.000	0.164	0.068	0.016	0.345	0.061	0.000	0.188	0.070	0.008
**Minority**	0.00216	0.021	0.918	0.135	0.080	0.092	-0.0718	0.223	0.748	0.264	0.122	0.031
**Rural residence**	-0.0269	0.023	0.251	0.147	0.079	0.062	0.110	0.115	0.338	0.122	0.095	0.197
**Proportion of boys/all surviving**	-0.148	0.024	0.000	-0.0656	0.083	0.428	-0.186	0.074	0.012	-0.0962	0.064	0.131
**Maternal education**												
no education				Ref			Ref					
Primary				-0.154	0.107	0.151	0.110	0.177	0.536			
secondary/higher				-0.256	0.141	0.070	0.00188	0.100	0.985			
**Marital status**												
Married				Ref			Ref					
living together				0.00994	0.122	0.935	-0.389	0.843	0.645			

	India 2005			Kenya 2014			Kyrgyz Republic 2012			Malawi 2010		
	Beta	SE	P-value	Beta	SE	P-value	Beta	SE	P-value	Beta	SE	P-value
divorced/not living together				-0.576	0.142	0.000	-1.485	0.309	0.000			
**Age**												
15-19							Ref					
20-24							-0.564	0.385	0.143			
25-29							-0.958	0.382	0.012			
30-34							-1.521	0.387	0.000			
35-39							-1.717	0.390	0.000			
**Wealth quintile**												
Lowest							Ref			Ref		
Low							0.0489	0.090	0.586	-0.116	0.069	0.091
Middle							-0.0549	0.087	0.530	-0.139	0.066	0.036
High							-0.126	0.104	0.227	-0.286	0.075	0.000
Highest							-0.0319	0.146	0.827	-0.273	0.098	0.005
Observations	520322			61702			52202			90441		
PH test P-value	0.296			0.263			0.083			0.800		
Stratified by	Age category, marital status, wealth quintile, maternal education			Age category, wealth quintile						Age category, maternal education, marital status		
Dropped because of collinearity							Partner's education					

	Mali 2012			Moldova 2005			Mozambique 2011			Namibia 2013		
	Beta	SE	P-value	Beta	SE	P-value	Beta	SE	P-value	Beta	SE	P-value
**IPV**	-0.00638	0.058	0.913	-0.0127	0.136	0.926	0.0649	0.053	0.224	1.191	0.689	0.084
**Maternal education**												
no education	Ref			Ref						Ref		
Primary	0.00672	0.095	0.943	0.239	0.763	0.754				0.0326	0.269	0.904
secondary/higher	-0.117	0.128	0.363	0.399	0.680	0.558				-0.207	0.279	0.457
**Marital status**												
married	Ref			Ref						Ref		
living together	0.222	0.262	0.397	0.452	0.232	0.052				-0.332	0.146	0.023
divorced/not living together	-0.784	0.541	0.147	-0.0989	0.238	0.678				-0.969	0.421	0.021
**Partner's education**												
no education	Ref			Ref			Ref			Ref		
Primary	0.203	0.099	0.041	-0.353	0.591	0.550	-0.102	0.064	0.110	-0.463	0.225	0.040
secondary or higher	-0.0458	0.110	0.677	-1.000	0.417	0.017	-0.185	0.094	0.048	-0.665	0.211	0.002
**Age first**												

	Mali 2012			Moldova 2005			Mozambique 2011			Namibia 2013		
	Beta	SE	P-value	Beta	SE	P-value	Beta	SE	P-value	Beta	SE	P-value
**cohabit**												
<20	Ref						Ref					
>=20	0.152	0.070	0.029				-0.0516	0.057	0.365			
**Minority**	0.0650	0.118	0.582	0.296	0.817	0.718	-0.268	0.228	0.239	0.0527	0.165	0.750
**Wealth quintile**												
Lowest	Ref									Ref		
Low	0.00980	0.085	0.908							0.323	0.191	0.092
Middle	0.0558	0.088	0.525							0.222	0.237	0.350
High	0.0527	0.093	0.570							0.439	0.234	0.061
Highest	-0.156	0.123	0.205							0.366	0.272	0.178
**Rural residence**	0.0402	0.096	0.675	0.000417	0.143	0.998	-0.0417	0.071	0.555	0.317	0.158	0.045
**Proportion of boys/all surviving**	-0.133	0.075	0.076	0.00130	0.120	0.991	-0.00291	0.066	0.965	0.0436	0.177	0.805
**IPVXlntime**										-0.390	0.217	0.073
**Age**												
15-19										Ref		
20-24										0.149	0.420	0.722
25-29										0.137	0.412	0.740
30-34										-0.0305	0.419	0.942
35-39										-0.309	0.428	0.469
Observations	54865			29340			78937			16255		
PH test P-value	0.940			0.514			0.991			0.148		
Stratified by		Age category			Age category, wealth quintile			Age category, marital status, wealth quintile, maternal education				
Dropped because of collinearity					Age at first cohabitation						Age at first cohabitation	

	Nepal 2011			Nigeria 2013			Pakistan 2012			Peru 2012		
	Beta	SE	P-value	Beta	SE	P-value	Beta	SE	P-value	Beta	SE	P-value
**IPV**	0.0292	0.096	0.761	0.398	0.184	0.030	0.129	0.065	0.047	0.484	0.290	0.095
**Partner's education**												
no education	Ref			Ref			Ref			Ref		
Primary	-0.156	0.118	0.187	0.0532	0.036	0.144	-0.0331	0.093	0.721	-0.110	0.195	0.571
secondary or higher	-0.279	0.121	0.021	0.0372	0.038	0.329	-0.101	0.077	0.190	-0.101	0.195	0.605
**Age first cohabit**												
<20	Ref			Ref			Ref					
>=20	0.135	0.125	0.280	0.139	0.031	0.000	0.305	0.069	0.000			

	Nepal 2011			Nigeria 2013			Pakistan 2012			Peru 2012		
	Beta	SE	P-value	Beta	SE	P-value	Beta	SE	P-value	Beta	SE	P-value
**Minority**	0.275	1.337	0.837	-0.186	0.083	0.025	0.0946	0.069	0.173			
**Wealth quintile**												
Lowest	Ref						Ref			**Ref**		
Low	-0.509	0.114	0.000				-0.157	0.088	0.074	-0.373	0.075	0.000
Middle	-0.565	0.129	0.000				-0.245	0.103	0.017	-0.572	0.098	0.000
High	-0.465	0.140	0.001				-0.228	0.106	0.031	-0.653	0.112	0.000
Highest	-1.155	0.183	0.000				-0.338	0.132	0.010	-0.714	0.142	0.000
**Rural residence**	-0.0293	0.123	0.812	-0.00902	0.031	0.772	0.0801	0.070	0.254	-0.298	0.072	0.000
**Proportion of boys/all surviving**	-0.233	0.105	0.026	0.0104	0.031	0.736	-0.197	0.081	0.015	-0.129	0.066	0.049
**IPVXlntime**				-0.109	0.058	0.060				-0.187	0.093	0.044
**Maternal education**												
no education							Ref			Ref		
Primary							0.0270	0.097	0.781	-0.424	0.134	0.002
secondary/higher							-0.0316	0.088	0.721	-0.506	0.141	0.000
**Marital status**												
Married							Ref			Ref		
divorced/not living together							-1.077	0.767	0.160	-0.504	0.131	0.000
**Age**												
15-19										Ref		
20-24										-0.0148	0.141	0.916
25-29										-0.233	0.141	0.099
30-34										-0.419	0.144	0.004
35-39										-0.466	0.150	0.002
living together										-0.00473	0.063	0.940
Observations	38866			313608			43561			173614		
PH test P-value	0.161			0.317			0.711			0.416		
Stratified by	Age category, maternal education			Age category, maternal education, marital status, wealth quintile			Age category					
Dropped because of collinearity	Marital status									Age at first cohabitation, minority status		

	Rwanda 2005			Sierra Leone 2013			Tajikistan 2012			Tanzania 2010		
	Beta	SE	P-value	Beta	SE	P-value	Beta	SE	P-value	Beta	SE	P-value
**IPV**	0.121	0.067	0.069	0.644	0.388	0.097	0.0705	0.073	0.332	-0.0289	0.051	0.575

	Rwanda 2005			Sierra Leone 2013			Tajikistan 2012			Tanzania 2010		
	Beta	SE	P-value	Beta	SE	P-value	Beta	SE	P-value	Beta	SE	P-value
**Maternal education**												
no education	Ref			Ref			Ref					
Primary	0.0574	0.075	0.446	0.0791	0.086	0.356	0.330	0.246	0.180			
secondary/higher	-0.0585	0.149	0.695	-0.0652	0.113	0.563	0.209	0.219	0.341			
**Partner's education**												
no education	Ref			Ref			Ref			Ref		
Primary	0.0869	0.075	0.246	0.116	0.103	0.258	0.669	0.581	0.249	-0.0474	0.068	0.483
secondary or higher	0.0755	0.125	0.545	-0.0504	0.082	0.540	0.0613	0.556	0.912	0.00963	0.104	0.926
**Age first cohabit**												
<20	Ref			Ref			Ref			Ref		
>=20	0.146	0.069	0.036	0.217	0.075	0.004	0.278	0.064	0.000	0.0604	0.056	0.281
**Proportion of boys/all surviving**	-0.0429	0.079	0.588	0.0338	0.082	0.680	-0.162	0.077	0.036	0.00826	0.067	0.902
**IPVXlntime**				-0.207	0.119	0.083						
**Minority**				-0.00461	0.084	0.956						
**Marital status**												
Married							Ref			Ref		
living together							-0.143	0.709	0.840	-0.749	0.146	0.000
divorced/not living together							-0.853	0.379	0.024	-0.526	0.103	0.000
**Wealth quintile**												
Lowest							Ref			Ref		
Low							-0.000825	0.103	0.994	-0.00842	0.068	0.901
Middle							-0.00406	0.103	0.968	-0.0311	0.072	0.666
High							-0.198	0.104	0.057	-0.290	0.080	0.000
Highest							-0.225	0.122	0.065	-0.616	0.122	0.000
**Rural residence**							0.0293	0.089	0.743	-0.0110	0.087	0.899
Observations	45583			62528			48890			83682		
PH test P-value	0.113			0.812			0.377			0.704		
Stratified by	Age category, rural residence, marital status, wealth quintile			Age category, rural residence, marital status, wealth quintile			Age category			Age category, maternal education		
Dropped because of collinearity	Minority status						Minority status			No minority		

	Timor Leste 2009			Ukraine 2007			Uganda 2011			Zambia 2013		
	Beta	SE	P-value	Beta	SE	P-value	Beta	SE	P-value	Beta	SE	P-value
**IPV**	0.634	0.379	0.094	-0.0156	0.294	0.958	0.0463	0.078	0.554	0.418	0.202	0.038
**IPVXlntime**	-0.223	0.121	0.066							-0.125	0.062	0.043
**Age**												
15-19	Ref			Ref			Ref					
20-24	-0.203	0.253	0.423	-0.500	0.515	0.332	-0.222	0.169	0.191			
25-29	-0.778	0.248	0.002	-0.782	0.495	0.114	-0.344	0.167	0.040			
30-34	-0.973	0.251	0.000	-1.000	0.485	0.039	-0.509	0.181	0.005			
35-39	-1.196	0.254	0.000	-0.889	0.521	0.088	-0.661	0.196	0.001			
**Partner's education**												
no education	Ref			Ref			Ref			Ref		
Primary	0.0553	0.097	0.570				0.0454	0.146	0.756	0.0681	0.070	0.328
secondary or higher	0.0561	0.094	0.552	31.00	1.176	0.000	-0.148	0.166	0.372	-0.0136	0.072	0.850
**Age first cohabit**												
<20	Ref			Ref			Ref			Ref		
>=20	0.178	0.078	0.022	0.472	0.184	0.010	0.196	0.098	0.044	0.106	0.043	0.014
**Minority**	-0.0833	0.195	0.669	0	.	.	0.0193	0.200	0.923	-0.124	0.135	0.359
**Rural residence**	0.00125	0.094	0.989	0.368	0.236	0.119	0.302	0.128	0.018	0.143	0.047	0.002
**Proportion of boys/all surviving**	-0.199	0.100	0.046	-0.0105	0.203	0.959	0.0321	0.103	0.755	-0.0353	0.047	0.457
**Marital status**												
Married				Ref			Ref					
living together				0.148	0.299	0.620	-0.0167	0.080	0.833			
divorced/not living together				-0.485	0.351	0.167	-0.892	0.192	0.000			
**Wealth quintile**												
Lowest				Ref			Ref					
Low				-0.348	0.241	0.149	0.00884	0.109	0.935			
Middle				-0.422	0.272	0.122	0.0170	0.120	0.887			
High				-0.668	0.345	0.053	0.0463	0.131	0.724			
Highest				-0.529	0.354	0.135	-0.111	0.169	0.510			
**Maternal education**												
no education							Ref			Ref		
Primary							0.0493	0.118	0.676	-0.116	0.052	0.025
secondary/higher							-0.0175	0.165	0.916	-0.208	0.065	0.001
Observations	33289			14662			26322			163664		
PH test P-	0.707			0.207			0.116			0.883		

	Timor Leste 2009			Ukraine 2007			Uganda 2011			Zambia 2013		
	Beta	SE	P-value	Beta	SE	P-value	Beta	SE	P-value	Beta	SE	P-value
value												
Stratified by	Maternal education, marital status, wealth quintile									Age category, marital status, wealth quintile		
Dropped because of collinearity												

	Zimbabwe 2010		
	Beta	SE	P-value
**IPV**	0.802	0.352	0.023
**IPV**XlntimeXlntime	-0.216	0.105	0.040
no education	Ref		
primary	0.0154	0.232	0.947
secondary/higher	-0.0274	0.233	0.906
no education	Ref		
primary	-0.559	0.203	0.006
secondary or higher	-0.526	0.201	0.009
**Minority**	-0.260	0.231	0.261
**Rural residence**	0.0545	0.106	0.608
**Proportion of boys/all surviving**	-0.128	0.077	0.094
Observations	73038		
PH test P-value	0.634		
Stratified by	Age category, marital status, wealth quintile		
Dropped because of collinearity	Age at first cohabitation		
