# Supplementary material for: Intimate partner violence and pregnancy spacing: results from a meta-analysis of individual participant time-to-event data from 29 low-and-middle-income countries
Source: BMJ Glob Health. 2018 Jan 13;3(1):e000304. doi: 10.1136/bmjgh-2017-000304 (PMC5859805; doi:10.1136/bmjgh-2017-000304)
Supplement: Supplementary data [file bmjgh-2017-000304supp002.pdf]

Figure S2. Assessment of proportional hazards assumption for one-stage meta-analysis of unintended pregnancies that resulted in live births

We interacted confounders that violated the proportional hazards (PH) assumption with a restricted cubic spline of time. For the one-stage meta-analysis of the relation between intimate partner violence (IPV) and time-to-unintended pregnancy, we assessed the Cox PH assumption for IPV and for the proportion of surviving children who were male (proportion of surviving male children over total surviving children), the only variables that were not interacted with time, by graphing the scaled Schoenfeld residuals against time. The Schoenfeld residuals compare the observed and expected predictor values where expected refers to the expected value for individuals still in the risk set when individual *i* experiences the event. A non-random pattern in the plot of the Schoenfeld residuals and observed event times is indicative of a violation of the PH assumption [1]. We did not find evidence for a non-random pattern in the plot of the Schoenfeld residuals for women's ever experience of IPV or for the proportion of surviving children who were male.

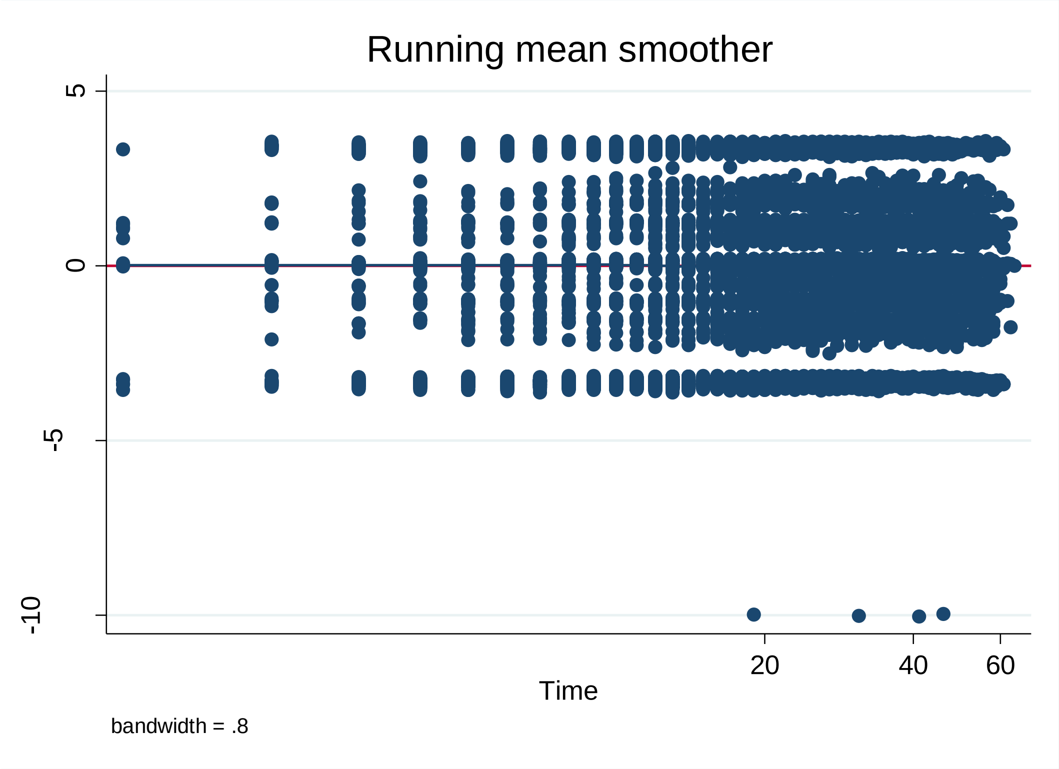

Figure S2. Plot of the scaled Schoenfeld residuals versus time to evaluate the PH assumption for women's ever experience of any form of IPV (emotional, physical, sexual) and for the proportion of surviving children who were male (a time-varying record of the number of surviving children over the total number of surviving children). The one-stage Cox PH shared frailty models for all incident pregnancies included an interaction between a restricted cubic spline with two knots for time and age (modelled as a restricted cubic spline with two knots), marital status, maternal education, partner's

education, household wealth quintile, rural residence; and country-level frailty terms to account for unmeasured, country-level factors.

References

1. Bradburn MJ, Clark TG, Love SB, Altman DG: **Survival Analysis Part III: Multivariate data analysis - choosing a model and assessing its adequacy and fit**. *Br J Cancer* 2003, **89**(4):605-611.
