# Supplementary material for: Intimate partner violence and pregnancy spacing: results from a meta-analysis of individual participant time-to-event data from 29 low-and-middle-income countries
Source: BMJ Glob Health. 2018 Jan 13;3(1):e000304. doi: 10.1136/bmjgh-2017-000304 (PMC5859805; doi:10.1136/bmjgh-2017-000304)
Supplement: Supplementary data [file bmjgh-2017-000304supp005.pdf]

Table S4. Period-specific hazard ratios for relation between intimate partner violence and time-to-incident pregnancy and time-to-unintended pregnancy

	**Shared frailty Cox PH for all incident pregnancies***		**Shared frailty Cox PH for unintended pregnancies that resulted in live births***	
**Number of months post resolution of most recent pregnancy**	**Intimate partner violence†**	**Number of surviving boys/total surviving children**	**Intimate partner violence†**	**Number of surviving boys/total surviving children**
	**HR (95% CI)**	**HR (95% CI)**	**HR (95% CI)**	**HR (95% CI)**
**0-3 months**	1.34 (1.13, 1.55)	0.77 (0.64, 0.92)	1.77 (1.30, 2.39)	0.77 (0.54, 1.10)
**4-6 months**	1.18 (1.07, 1.31)	0.99 (0.89, 1.10)	1.40 (1.17, 1.67)	1.21 (0.99, 1.48)
**7-9 months**	1.20 (1.10, 1.31)	0.95 (0.87, 1.04)	1.45 (1.25, 1.68)	1.01 (0.86, 1.20)
**10-12 months‡**, §	1.00 (0.93, 1.09)	0.95 (0.88, 1.03)	1.15 (1.01, 1.31)	0.98 (0.85, 1.13)
**13-15 months**	1.07 (1.00, 1.14)	0.99 (0.92, 1.06)	1.25 (1.19, 1.31)	0.96 (0.91, 1.02)
**16-18 months**	1.15 (1.09, 1.22)	0.94 (0.88, 1.00)	1.45 (1.30, 1.61)	1.02 (0.89, 1.15)

CI, confidence interval; HR, hazard ratio

*Adjusted for age (modelled as a restricted cubic spline with two knots), marital status, maternal education, partner's education, household wealth quintile, rural residence; conditional on country-level frailty terms

† Includes emotional, physical, and/or sexual violence

‡The analysis of all incident pregnancies includes an interaction between analysis time (modelled as a restricted cubic spline with two knots) and rural residence

§The analysis of all unintended pregnancies that result in live births includes an interaction between analysis time (modelled as a restricted cubic spline with two knots) and household wealth quintiles and between analysis time and rural residence

The hazard ratio is dependent on the duration of follow-up. While period-specific hazard ratios may provide more information than the average hazard ratio for the entire follow-up period, period-specific hazard ratios have a built in selection bias[1]: the estimation of the hazard ratio for a given period is limited to women who survived (ie. did not have an incident pregnancy) up until the beginning of that period.

References

1. Hernán MA. The hazards of hazard ratios. *Epidemiology (Cambridge, Mass)* 2010;21(1):13.
