# Supplementary material for: Intimate partner violence and pregnancy spacing: results from a meta-analysis of individual participant time-to-event data from 29 low-and-middle-income countries
Source: BMJ Glob Health. 2018 Jan 13;3(1):e000304. doi: 10.1136/bmjgh-2017-000304 (PMC5859805; doi:10.1136/bmjgh-2017-000304)
Supplement: Supplementary data [file bmjgh-2017-000304supp001.pdf]

Figure S1. Assessment of proportional hazards assumption for one-stage meta-analysis of all incident pregnancies

We interacted confounders that violated the proportional hazards (PH) assumption with a restricted cubic spline of time. For the one-stage meta-analysis of intimate partner violence (IPV) and time-to-incident pregnancy, we assessed the Cox proportional PH assumption for the proportion of surviving male children over total surviving children, the only variable that was not interacted with time, by graphing the scaled Schoenfeld residuals against time. The Schoenfeld residuals compare the observed and expected predictor values where expected refers to the expected value for individuals still in the risk set when individual *i* experiences the event. A non-random pattern in the plot of the Schoenfeld residuals and observed event times is indicative of a violation of the PH assumption [1]. We did not find evidence for a non-random pattern in the plot of the Schoenfeld residuals for the proportion of surviving children who were male.

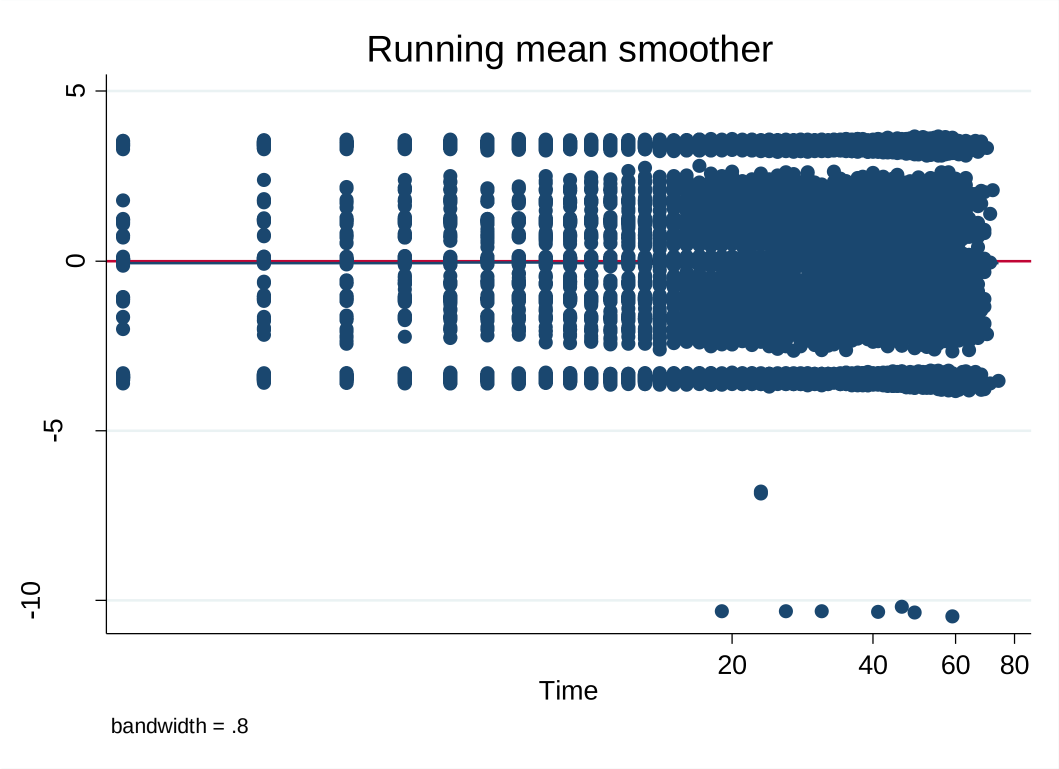

Figure S1. Plot of the scaled Schoenfeld residuals versus time to evaluate the PH assumption for the proportion of surviving children who were male (a time-varying record of the number of surviving children over the total number of surviving children). The one-stage Cox PH shared frailty models for all incident pregnancies included an interaction between IPV and ln(time); an interaction between a restricted cubic spline with two knots for time and age (modelled as a restricted cubic spline with two knots), marital status, maternal education, partner's education, household wealth quintile, rural residence; and country-level frailty terms to account for unmeasured, country-level factors.

References

1. Singer JD, Willett JB: **Applied longitudinal data analysis: Modeling change and event occurrence**: Oxford university press; 2003.
